# Housing for People with an Acquired Brain or Spinal Injury: Mapping the Australian Funding Landscape

**DOI:** 10.3390/ijerph16162822

**Published:** 2019-08-07

**Authors:** Courtney J. Wright, Jacinta Colley, Kate Knudsen, Elizabeth Kendall

**Affiliations:** The Hopkins Centre, Griffith University, Meadowbrook, Queensland 4131, Australia

**Keywords:** National Disability Insurance Scheme, National Injury Insurance Scheme, disability, independence, rehabilitation, brain injury, spinal cord injury, housing, support, policy

## Abstract

This research aimed to synthesize housing supports funded by 20 major insurance-based schemes for Australians with an acquired brain injury (ABI) or spinal cord injury (SCI). Publicly available grey literature (i.e., primary information from respective scheme websites) was systematically reviewed and compared. There were notable differences between the different scheme types (disability vs. workers compensation schemes) and across different States. Collectively, scheme funding was more likely to be focused on housing infrastructure and service delivery, than on tenancy support. Australians who are least likely to benefit from the current funding context are those whose home cannot be reasonably modified, are wanting to build or purchase a new home, do not have suitable, alternative short- or long-term housing options if their current home is not feasible, require support to maintain occupancy of their home or financial assistance to move into a new home, may benefit from case management services, family supports, and assistance animals, and/or cannot afford their rent or home loan repayments. Several interactions, inconsistencies, contradictions, and gaps that warrant further attention were also revealed. This review has highlighted the need for policy makers to provide transparent information about housing entitlements for individuals with ABI or SCI, and their families. A unified, evidence-based framework to guide the funding of housing and housing support services may increase the consistency of interventions available to people with ABI or SCI and, therefore, improve outcomes.

## 1. Introduction

It is widely acknowledged that housing is a social determinant of health and wellbeing [1,2,3,4,5,6,7]. Causal associations between suitable housing environments and positive physical and mental health, and inappropriate housing environments and poor physical and mental health, has transcended a focus on basic housing and sanitary conditions to more complex direct and indirect influencers; including housing policy, housing location, neighborhood characteristics (i.e., physical accessibility; socioeconomic conditions), affordability, tenancy (i.e., who people live with), tenure security, and access to social and employment networks, educational opportunities, transportation systems, services (i.e., shopping; banking; health care facilities) and public resources (i.e., parks; museums; libraries) [1,2,3,4,5,6,7]. Further, causal associations between housing and health/wellbeing have been linked to multiple population groups; for example, children [8,9], adults [10,11], people from developed and developing nations [12], different geographical areas (i.e., urban, regional, remote locations) [11,13], a range of cultures [14,15], able-bodied populations [8,10], and people with diverse health conditions and impairments [16,17]. Despite this well-established, multifaceted link between housing and health/wellbeing, individuals with an acquired brain injury (ABI) or spinal cord injury (SCI) incur significant housing challenges [18,19,20].

As a result of the physical, cognitive, sensory, perceptual, communicative, and/or behavioral consequences directly related to their neurological injury [21,22], individuals with ABI and/or SCI often require specialist support to live as independently as possible at home and participate in their communities. These supports may include physically accessible environments, access to specialized equipment, assistance with activities of daily living and personal care, and/or support to access and participate in community activities [3]. Unfortunately, many people with ABI and SCI are forced to reside in unsuitable residential environments (e.g., an impractical dwelling; an impractical neighborhood; group home with strict routines; residential aged care), or housing that is not their preferred option (e.g., family home with aging parents), due to broader and persistent issues relating to housing availability and housing suitability [23,24,25,26,27,28]. Living in unsuitable residential environments without access to the degree of specialist housing support needed by individuals with ABI and SCI directly impacts the person’s wellbeing and quality of life, and that of their family who often adopt an informal caring role [1,3,18,19,29,30,31].

Several personal and systemic factors have contributed to the current housing landscape for people with ABI and SCI. These factors include pre-existing personal factors (e.g., debts associated with higher education; potentially low amounts of savings), post-injury personal factors (e.g., low income due to limited or no participation in work; high housing costs associated with their disability, such as home modifications), and systemic factors (e.g., lack of physically accessible private housing; shortage in funding services to support people with ABI or SCI to live in their own home and participate in their community) [29,30,31]. These complex and multifaceted factors result in many individuals with ABI and SCI being unable to afford the specialized support they require to address their housing needs. People with ABI or SCI may therefore rely on insurance schemes to provide financial assistance so that they may access the housing support they require.

In Australia, people with ABI or SCI may be eligible for financial assistance (funding) through national or State/Territory-based insurance schemes. Major schemes across Australia include the National Disability Insurance Scheme (NDIS), National Injury Insurance Scheme (NIIS), workers compensation schemes, and Compulsory Third-Party (CTP) insurance schemes. (This research is focused on the major insurance schemes available to Australians with ABI and SCI. However, it is important to note that individuals may be able to apply and receive housing support from other schemes run by non-profit agencies and community services). Appendix A provides an overview of these major schemes as they relate to each Australian State and Territory. A ‘person-centered’ philosophy underpins these major schemes, where the person with disability is placed at the center of decision making regarding the supports and services they use [32] Supports and services may relate to housing, education, employment, social participation, independence, and/or health [33]. Rather than traditional ‘block’ funding arrangements where the Government provides grants to approved providers to deliver community service programs, individualized funding is apportioned to individuals with disability through a person-centered planning process [32]. In this way, the current funding landscape in Australia is intended to assist individuals with disability to improve their situation (including housing) by providing financial assistance in a way that maximizes consumer choice. The NDIS has been operational in trial sites across Australia since 2013. The full roll out of the NDIS and NIIS schemes commenced in July 2016, and is expected to be fully operational across the country by December 2019. Thus, this is a transition period for all national and State/Territory-based Schemes as they determine their roles and responsibilities following the most significant policy reform in Australia’s history.

Given the crucial role of insurance schemes in contributing to housing improvements for individuals with ABI and SCI, this research aimed to review and synthesize publicly available information about housing supports funded by the major Australian insurance schemes for people with ABI or SCI. It is anticipated that the findings of this review will provide individuals with ABI or SCI, and their families, an overview of available funding for housing supports across Australia (the authors plan to communicate the review findings in plain English and Easy Read summaries on The Hopkins Centre website: https://www.hopkinscentre.edu.au/). It is also envisaged that the findings will enable comparison of funded supports across the diverse schemes, and on a national scale, to inform future decision making in this area.

## 2. Method

A scoping review of available information was systematically conducted. The scoping review method is considered appropriate for addressing broad research questions and when assessment of study designs and quality may not be required [34]. In accordance with the methodological framework described by Arksey and O’Malley [34], five key steps were completed: (1) identify the research question/s; (2) identify relevant literature; (3) study selection; (4) chart the data; and (5) collate, summarize and report the results. Each step is described below.

### 2.1. Step One: Identify the Research Question/s

To address the review aim, the following research questions were proposed:

1. What do the major Australian insurance schemes fund in relation to housing for a person with ABI or SCI?
2. What individual factors impact on the availability of housing supports in each scheme (i.e., type of injury and level of severity; person’s age; location; family contexts; existence of other mental or physical health conditions)?

For the purpose of this review, funding related to housing was broadly defined as funding that supported people to live as independently as possible in their home and participate in their community. This definition included funding for infrastructure (e.g., home modifications), tenancy (e.g., assistance with rent or mortgage repayments), and service delivery (e.g., attendant care; assistance with accessing and participating in the community) [3]. Funding for hospital or medical care, rehabilitation services, and vocational and educational support services (including travel to any such services) was beyond the scope of the current review. Funding related to living outside of Australia, financial assistance in the form of regular payments (e.g., income supplement), or historical compensation arrangements (e.g., superseded legislation) were also out of scope.

### 2.2. Step Two and Step Three: Identify Relevant Literature and Study Selection

The relevant Australian schemes were first identified and their websites were systematically searched for relevant information. Sources of information reviewed included:

- Information detailed on scheme websites;
- Online information sheets and resources produced by scheme authorities (e.g., fact sheets, policy statements, and scheme guidelines);
- Scheme annual reports;
- Case studies provided by each scheme; and
- Relevant State and Commonwealth legislation referred to by schemes.

To provide an analysis of the existing funding context, and how national and interstate insurance schemes interact, only current information detailed on scheme websites and resources published after the introduction of the National Injury Insurance Scheme in 2016 (e.g., legislation; annual reports) were reviewed. No other limitations were applied to the searches.

### 2.3. Search Strategy

The search strategy and data extraction process employed in this study was “systematic, transparent and reproducible” [35]. Two researchers independently and systematically searched the scheme websites in June 2018 for relevant information. All sources of information were noted, and the details of relevant sources of information were recorded for further examination and data extraction (a record of resources reviewed is available at: https://research-storage.griffith.edu.au/owncloud/index.php/s/b58UYegujruvsd5). Only information that addressed the research questions was included in the review. Any discrepancy between the two researchers was discussed with a third researcher to achieve consensus.

### 2.4. Step Four: Chart the Data

Data extraction was performed independently by two researchers. A standard data extraction form was used to provide consistency and structure to the data extraction process. Data extracted from relevant sources included: (a) scheme title; (b) the national or interstate focus of the scheme (and Australian State/Territory of relevance, if applicable); (c) citation details; (d) funded housing supports in relation to infrastructure, tenancy, and service delivery; and (e) influencing individual factors, as available. Any discrepancy between the two researchers was reconciled by mutual agreement with a third researcher.

### 2.5. Step Five: Collate, Summarize and Report the Results

A narrative synthesis was used to describe the data extracted from the relevant sources. The data was synthesized at the level of: (a) individual funding schemes; (b) collective national and State/Territory-based schemes; (c) housing funding contributions (e.g., infrastructure, tenancy, and service delivery supports); and (d) an overall summary. Quality appraisal of data sources was not relevant for this scoping review [34].

## 3. Results

This review identified 20 major insurance schemes across Australia that currently provide housing-related funding to people with ABI or SCI. This included four national schemes (i.e., the NDIS, Seacare, Comcare, and the Department of Veterans’ Affairs [DVA] Compensation Scheme) and 16 State/Territory-based schemes inclusive of the NIIS and workers compensation schemes. CTP insurance schemes (where still occurring across Australia) were not included in this review since most individuals seriously injured in a motor vehicle accident (post-2016) will access housing or housing support through a lifetime care insurance scheme such as NIIS. Although CTP schemes (common law compensation payments) have historically included a component for housing modifications and care to live independently, they now predominantly focus on funding non-housing related supports for individuals with serious ABI or SCI (e.g., economic loss; expenses for treatment related to the person’s injury; pain and suffering).

Table 1 provides an overview of the funding contributions of the 20 relevant schemes in relation to three main categories of housing support (i.e., infrastructure, tenancy, and service delivery) following ABI or SCI. Particular infrastructure, tenancy, and service delivery supports that are funded (or not funded) by respective schemes, as well as demographic factors that may influence the availability of funding to individuals, are outlined following this summary. Scheme funding contributions toward housing supports did not differ between ABI and SCI conditions.

### 3.1. Housing Infrastructure Funding

In relation to housing infrastructure, funding across the 20 schemes related to seven subcategories. These subcategories included home modifications, new builds owned by individuals, homes purchased by individuals, consumables and equipment, vehicle modifications, and short- and long-term accommodation. Table 2 provides a summary of particular infrastructure supports funded (or not funded) by the schemes and highlights the differences between schemes based on publicly available information. Investment in housing infrastructure varied dramatically with a focus on supporting individuals to remain living in their current housing rather than move into another home that might better address the person’s needs. For example, all 20 (100%) schemes funded home modifications (although the type and extent of modifications financed differed across schemes) whereas only three (15%) schemes contributed funding for new builds owned by individuals, one (5%) scheme supported the purchase of a new home owned by individuals, and six (30%) schemes provided funding in relation to other long-term housing options.

### 3.2. Tenancy Funding

In relation to tenancy, funding across the 20 schemes related to three subcategories that focused on supporting individuals to find, move into, and/or maintain occupancy of a private or rental home. These subcategories included rent assistance, support obtaining and/or maintaining a tenancy, and relocation costs. Table 3 provides an overview of tenancy-related support funded by respective schemes. Only one (5%) scheme reported the availability of funding for rent assistance, and six (30%) schemes reported the availability of support to move into another home. There was no mention of assistance with mortgage repayments for individuals who were injured after purchasing their home or people with ABI or SCI wanting to enter the property ownership market. There was a general lack of publicly available information regarding tenancy funding, which is likely problematic given the increased risk of individuals with ABI or SCI residing in unsuitable residential environments or housing that is not their preferred option [23], together with the potential for individuals to have low income following injury [29,30,31] and thus not be in a strong position to change their housing situation.

### 3.3. Service Delivery Funding

Service delivery funding across the 20 schemes related to six subcategories that focused on supporting individuals to live an ordinary life once established in their home. These subcategories included attendant (personal) care, household services, community access and participation, case management services, family supports, and assistance animals. Table 4 provides a summary of particular service delivery supports funded (or not funded) by the schemes and outlines the differences between schemes based on publicly available information. The majority of schemes provided funding for attendant (personal) care (n = 17; 85%) and household services (n = 17; 85%). Fewer schemes supported individuals to access and participate in their community (n = 11; 55%), increase the capacity and capability of informal supports such as the person’s family (n = 11; 55%), and increase the person’s independence through assistance animals (n = 4; 20%).

### 3.4. Demographic Influencing Factors

Demographic factors that influenced the general provision of housing-related funding for Australians with ABI or SCI were identified using the publicly available eligibility criteria. These demographic factors included the person’s age, gender, location, culture, degree of functioning, intrinsic characteristics, whether the person is in receipt of external compensation, and whether the person has a pre-existing medical condition or comorbidities. These influencing factors raise several challenges because unbiased and reliable assessment is so complex. Table 5 provides a summary of these influencing factors.

## 4. Discussion

This scoping review aimed to synthesize the publicly available information about housing supports funded by the major Australian insurance schemes for people with ABI or SCI. Funded supports related to housing were compared across the diverse schemes, and between different Australian States and Territories, to produce an overall snapshot that would inform future decision making in this area. The extent of missing information across the schemes made it difficult to draw firm conclusions.

There were notable differences in available information across the different schemes. For example, the disability-related schemes (i.e., NDIS and NIIS schemes) generally provided more information about funded housing supports for people with an ABI or SCI than the workers compensation schemes. This finding is not surprising given the focus of disability-related schemes on supporting individuals to live ordinary lives compared to the particular focus of workers compensation schemes in assisting an injured person to return to work. The extent of missing information from the workers compensation schemes makes it difficult to confirm the range of housing supports likely to be funded by these schemes for people with an ABI or SCI. It is important to note that there is strong research evidence regarding the role of suitable housing and transportation systems in supporting return to work following an acquired injury; particularly since inadequate transportation has been identified as a significant barrier for return to work and individuals with ABI or SCI may participate in home-based employment [1,183,184,185,186,187]. Since participation in work is considered an important indicator of social integration [186,187], and being employed is associated with increased self-esteem, socialization, physical activity (and therefore fewer medical treatments), adjustment to injury, life satisfaction, wellbeing and financial independence [186,187,188,189], an expanded focus on housing in all schemes might, therefore, be justified.

At a national level, the NDIS and all Victorian schemes provided more information about funded housing supports for people with an ABI or SCI compared to other national schemes (Seacare, Comcare, and the DVA scheme) and the remaining State and Territory schemes. It is important to note that the amount of information available does not correlate with the most comprehensive housing support. Rather, this finding suggests that people with ABI or SCI who are eligible for funding through the NDIS or Victorian schemes (and their families) may be more informed about their housing entitlements, and thus in a stronger position to advocate for appropriate housing supports.

There were also notable differences among the housing infrastructure, tenancy, and service delivery supports that were funded across the schemes. Funding was focused predominantly on housing infrastructure and service delivery, rather than tenancy support. For example, funding across the 20 schemes was consistently provided for home modifications (n = 20; 100%), consumables and equipment (n = 19; 95%), vehicle modifications (n = 18; 90%), attendant (personal) care (n = 17; 85%), and household services (n = 17; 85%). Less funding was provided for new builds (n = 3; 15%), home purchases (n = 1; 5%) and rent assistance (n = 1; 5%) with two, four, and seven schemes specifically stating that they would not fund these housing supports. However, it is important to note that these three supports had a high rate of missing information. That is, information was missing from more than 50% of schemes where the identified housing supports were not mentioned in any public documents, so it was not clear whether the respective schemes provided funding for these supports or not. Additional housing supports with high rates of missing information included short-term accommodation (information missing from 12 [60%] schemes), long-term accommodation (n = 14; 70% schemes), support obtaining and/or maintaining a tenancy (n = 14; 70% schemes), relocation costs (n = 14; 70% schemes), case management services (n = 16; 80% schemes), and assistance animals (n = 16; 80% schemes). Given the extent of this missing information across the 20 major insurance schemes, the nature of support for individuals with ABI or SCI to find short- or long-term accommodation, move into their new home, retain their home, and manage their community services and supports remains unclear.

Australians with ABI or SCI who are most likely to benefit from the existing schemes are those who reside in a home that can be modified and have access to attendant (personal) care, household services, and community access and participation support (although this does not guarantee that the modifications undertaken or support provided will meet all of the person’s needs). People least likely to benefit from the current funding landscape are those whose home cannot be reasonably modified, are wanting to build or purchase a new home, do not have suitable, alternative short- or long-term housing options if their current home is not feasible, require support to maintain occupancy of their home or financial assistance to move into a new home, may benefit from case management services, family supports, and assistance animals, and/or cannot afford their rent or home loan repayments. These are concerning findings when considering the pre-existing personal factors (e.g., debts associated with higher education; potentially low amounts of savings) and post-injury personal factors (e.g., low income due to limited or no participation in work; high housing costs associated with disability) that are commonly experienced by people with ABI and SCI [29,30,31].

### 4.1. Implications

Several important implications emerged from this comparison across schemes, including the identification of interactions, inconsistencies, contradictions, and gaps.

This review has highlighted several interactions that complicate the funding provided by schemes. For example, schemes will only provide funding for housing supports that are disability-related (e.g., the result of the person’s accident). Funding will not be provided if the person’s medical condition or comorbidities are pre-existing. How the connection between the person’s medical condition or comorbidities and the person’s accident is defined, determined, and judged by scheme authorities (i.e., what constitutes a medical condition or comorbidity; whether delayed onset is considered) was not explained within public documents. This ambiguity represents a major grey area within scheme funding guidelines. For instance, fitness declines rapidly for people with SCI [190] and chronic illness is experienced at a high rate in ABI and SCI populations, however onset might be delayed [191,192]. The absence of clear guidelines surrounding the person’s medical condition and comorbidities places the person at risk of piecemeal, rather than holistic, support. Likewise, whether funding is provided for pre-existing conditions or comorbidities that are exacerbated as a result of the person’s accident was not clarified. In addition, some schemes specified funding restraints that interact with external services (e.g., the provision of short-term accommodation for a specified time period while home modifications are completed). Whether the timeframes indicated are variable in response to external constraints (i.e., builders; building conditions; supply and demand) was not clear. Interactions within and across schemes, and also with external services and programs, differed across the schemes which can create discrepancies, confusion, and gaps.

The inconsistencies identified within and across schemes represent inequitable access to housing support. For example, some schemes fund partial or full costs for the provision of home exercise/gym equipment, climate control, and travel to recreational activities while other schemes do not fund these supports. How schemes decided supports like these are not disability-related, and therefore not funded, remains unclear. Deterioration following ABI and SCI can be reduced by exercise [193,194], complications caused by temperature regulation deficits within the ABI population can be minimized by climate control [195], and engagement in recreational activities following ABI and SCI can enhance the person’s psychosocial wellbeing and improve quality of life after injury [196,197]. Inequitable access to housing supports such as these can lead to disparity of outcomes. Indeed, national schemes such as the NDIS were intended to remedy previous inconsistencies between schemes and jurisdictions. In particular for people with ABI and/or SCI, how individuals acquired their disability determined the level of compensation they received more than how severe the person’s disability was. The NDIS was intended to supersede this previous funding context, and provide need-based support, but the process is ongoing and far from straight-forward.

Several contradictions within and across schemes were also identified. For example, repair and/or replacement of home modifications, equipment and vehicle modifications may be funded by schemes, however the cost of insurance for these supports may not be funded. This paradox is interesting given that insurance may reduce the cost of repair or replacement. Similarly, cosmetic and personalized fittings not deemed ‘reasonable and necessary’ (in relation to home modifications) are not funded by schemes, which contradicts the personalized and person-centered philosophy held by most schemes. How the boundary between personal preference and ‘reasonable and necessary’ is defined and enacted upon remains unclear. Additional contradictions within and across schemes may result in disincentives to improve one’s situation. For instance, assistance animals are highly trained disability support services that enable people with disabilities to safely and independently participate in daily activities [198]. Investment in assistance animals may replace the need for a funded support worker, however only four (20%) schemes provided funding for this support according to public documents. An expectation of at least one of these four schemes is that the assistance animal is fully trained, and so additional training is not funded. However, specific training is likely needed to suit the home and needs of the person, highlighting further contradictions by the schemes.

This review has also revealed a number of gaps that warrant further attention. These gaps may be characterized by a lack of a preventative approach (e.g., housing supports that promote ongoing rehabilitation or reduce decline or deterioration over time, such as swimming pools, hydrotherapy pools, spas, gym memberships and home modifications to support the natural ageing process of an individual, are not funded), and little recognition of broader social determinants of health [1] and health promotion [199] as an integral component of lifestyle (e.g., hobbies or personal lifestyle interests and housing supports that enable people to increase control over, and improve their health, such as case management services that assist the person to make informed decisions and exercise choice and control, are not funded by the majority of schemes). Systemic gaps were also identified. For example, many and various housing supports that were identified as being the responsibility of other departments and systems and thus not funded by the respective schemes (e.g., specific equipment; school teacher aides; hospital and GP visits; transport infrastructure including road and footpath infrastructure; homelessness prevention and outreach; access to temporary and long-term housing for people who are homeless or at risk of homelessness) may contribute to people with ABI and SCI ‘falling through the cracks’. This review has also highlighted a lack of recognition of change over time. Funding for some housing supports (e.g., further modifications to the same vehicle) may only be provided where there are ‘unforeseen and significant changes to the person’s needs’. However, how ‘unforeseen’ and ‘significant’ is defined was not described in any public documents, and whether deterioration or ageing are considered ‘unforeseen’ remains unclear. Additional complex concepts specified within scheme public documents with no clear understanding of how these concepts are assessed and acted upon include ‘age appropriate’, ‘no clear benefit’, and ‘maximum medical improvement’. This ambiguity may lead to funding decisions that are based on subjective judgements which could be potentially biased, stereotyped and not well-assessed at any one point in time.

### 4.2. Implications for Consumers and Policy Makers

The interactions, inconsistencies, contradictions, and gaps revealed by this review highlight several opportunities for consumers and policy makers. Individuals with ABI or SCI, and their families, may use this information to inquire about particular housing supports they may need to live as independently as possible at home and participate in the community, and that have not been mentioned in their respective scheme guidelines, fact sheets, or other public documents. The findings of this review may therefore empower individuals and their families to pursue information about housing supports they may be entitled to, in order to improve their housing situation. For policy makers, this review has highlighted equality issues that must be considered and acted upon, particularly if individuals with ABI or SCI are unable to access the housing support they require from other government or non-government schemes. In addition, the identification of missing information across the schemes by this review represents an opportunity for all schemes to provide transparent information about housing entitlements for individuals with an ABI or SCI, and their families. The development and communication of a unified, evidence-based framework for housing support that is tailored to ABI and SCI, and incorporates a lifelong rehabilitation approach that recognizes broader social determinants of health [1] and health promotion [199] as an integral component of lifestyle, may inform future decision making in this area. There is now adequate evidence to refute the notion of a recovery plateau following ABI and SCI, and thus great hope for long-term neurological recovery [200,201,202,203,204,205]. Such an approach would likely improve housing outcomes for consumers. Lastly, people with ABI and/or SCI are considered to be among the most supported people with disability in Australia due to their often high level of support need, and so the nature of housing support provided, and information available, for the many other disability types ought to be explored.

### 4.3. Limitations and Future Research Directions

The findings of this review must be interpreted in context of its limitations. First, the review findings reflected information made publicly available through each scheme’s official website. This included the availability of the resources themselves, as well as the breadth and depth of information provided in each resource. The potential of missing information was identified in relation to several schemes, meaning that the findings reflected only the available data. Despite this limitation, Arksey and O’Malley [34] argued that scoping reviews are often broad in scope and can identify gaps in knowledge requiring further examination. The findings of this scoping review can, therefore, inform subsequent investigations, including examination of: (a) actual housing funding across the schemes in comparison to policy; (b) decision-making processes that translate policy into housing provision and service delivery; and (c) the impact, utility and benefit of funded housing supports for individuals with ABI or SCI to inform future policy. The knowledge generated through this research could inform future, evidence-based decision making in this area.

## 5. Conclusions

This scoping review evaluated the publicly available information about housing supports funded by major Australian insurance schemes for people with an ABI or SCI. The 20 schemes included in this review were four national schemes (i.e., the NDIS, Seacare, Comcare, and DVA Compensation Scheme) and 16 State/Territory-based schemes inclusive of the NIIS and workers compensation schemes. This research highlighted distinct differences between the 20 schemes in terms of the level of detail available in their public documents, the extent and type of housing support each scheme would fund, and the demographic factors that influenced the availability of particular housing supports.

The interactions, inconsistencies, contradictions, and gaps revealed by this review highlight several opportunities for consumers and policy makers. There is a need for transparent information about housing entitlements for individuals with an ABI or SCI, and their families. There is also a need for policy makers to investigate ways in which different elements of their schemes interact with or contradict other elements or with other schemes, creating disincentives, discrepancies or gaps. The development and communication of a unified, evidence-based framework for housing support that is tailored to ABI and SCI may improve housing outcomes for consumers.

## Figures and Tables

**Table 1:** Overview of Housing Support Funding for Australians with an Acquired Brain or Spinal Cord Injury.

Major Australian Funding Schemes	Infrastructure							Tenancy			Service Delivery					
	Home Modifications	New Builds Owned by Individuals	Home Purchases Owned by Individuals	Consumables and Equipment	Vehicle Modifications	Short-Term Accommodation	Long-Term Accommodation	Rent Assistance	Support Obtaining and/or Maintaining Tenancy	Relocation Costs	Attendant (Personal) Care	Household Services	Community Access and Participation	Case Management Services	Family Supports	Assistance Animals
**National Schemes**																	
NDIS	**√**	**√**	^	**√**	**√**	**√**	**√**	X	**√**	**√**	**√**	**√**	**√**	**√**	**√**	**√**
Seacare	**√**	^	^	**√**	**√**	^	^	^	^	^	**√**	**√**	^	^	^	^
Comcare	**√**	^	^	**√**	**√**	^	^	^	^	^	**√**	**√**	**√**	^	^	^
DVA Compensation Scheme	**√**	^	^	**√**	**√**	^	**√**	X	**√**	^	**√**	**√**	**√**	**√**	**√**	**√**
**State/Territory-based Schemes**																	
**Queensland (QLD)**																	
NIIS: NIISQ	**√**	^	^	**√**	**√**	^	^	X	^	^	**√**	**√**	**√**	^	**√**	^
Workers Compensation: WorkCover (QLD)	**√**	^	^	**√**	**√**	^	^	^	^	^	^	^	**√**	^	^	^
**New South Wales (NSW)**																	
NIIS: icare Lifetime Care	**√**	^	X	**√**	**√**	**√**	^	X	**√**	**√**	**√**	**√**	**√**	^	**√**	^
Workers Compensation: icare Workers Care	**√**	^	^	**√**	**√**	^	^	^	^	^	**√**	**√**	^	^	^	^
**Australian Capital Territory (ACT)**																	
NIIS: Lifetime Care and Support Scheme	**√**	X	X	**√**	**√**	**√**	^	X	**√**	**√**	**√**	**√**	**√**	^	^	^
Workers Compensation (ACT)	**√**	^	^	**√**	^	^	^	^	^	^	^	^	^	^	^	^
**Victoria (VIC)**																	
NIIS: Transport Accident Compensation Scheme	**√**	**√**	X	**√**	**√**	**√**	**√**	X	**√**	**√**	**√**	**√**	**√**	**√**	**√**	**√**
Workers Compensation: WorkSafe (VIC)	**√**	**√**	**√**	**√**	**√**	**√**	**√**	**√**	**√**	**√**	**√**	**√**	**√**	**√**	**√**	^
**Tasmania (TAS)**																	
NIIS: Motor Accidents Insurance Scheme	**√**	^	^	**√**	**√**	**√**	**√**	^	^	^	**√**	**√**	^	^	**√**	^
Workers Compensation: WorkSafe (TAS)	**√**	^	^	**√**	**√**	^	^	^	^	^	**√**	**√**	^	^	^	^
**Northern Territory (NT)**																	
NIIS: Motor Accidents Compensation Scheme	**√**	^	^	**√**	**√**	**√**	^	^	^	^	**√**	**√**	^	^	^	^
Workers Compensation: WorkSafe (NT)	**√**	^	^	^	**√**	^	^	^	^	^	**√**	**√**	^	^	**√**	^
**South Australia (SA)**																	
NIIS: Lifetime Support Scheme	****√****	X	X	**√**	**√**	**√**	**√**	X	^	**√**	**√**	**√**	**√**	^	**√**	**√**
Workers Compensation: ReturnToWork (SA)	**√**	^	^	**√**	^	^	^	^	**√**	^	**√**	**√**	**√**	^	**√**	^
**Western Australia (WA)**																	
NIIS: Catastrophic Injuries Support Scheme	**√**	^	^	**√**	**√**	^	^	^	^	^	**√**	**√**	^	^	**√**	^
Workers Compensation: WorkCover (WA)	**√**	^	^	**√**	**√**	^	^	^	^	^	^	^	^	^	^	^

Note. Data was obtained from publicly available information. The 16 ‘infrastructure’, ‘tenancy’, and ‘service delivery’ subcategories were data-driven. √ = funding provided; X = funding not provided; ^ = no funding described (i.e., not clear whether funding provided or not). DVA = Department of Veterans’ Affairs; NDIS = National Disability Insurance Scheme; NIIS = National Injury Insurance Scheme; NIISQ = National Injury Insurance Scheme Queensland.

**Table 2:** A Comparison of Infrastructure Funding across the Schemes.

Subcategory	No. Schemes Funding Available	Eligibility Criteria	Description of Funded Supports	Description of Supports Not Funded	Differences across Schemes (if Applicable)
Home modifications	20 (100%)	A need for modifications based on a compensable condition [36,37,38,39,40,41,42,43,44,45] Home modifications required improve functioning, independence and/or safety [38,39,40,41,46,47,48,49,50,51,52,53,54,55,56] The person’s inability to use certain necessary facilities or areas within their home is permanent and assistance from carers and community services are inadequate to the purpose [38,39,40,41,42,43,46,47,48,49,50,51,52,53,54,55,56] The person’s primary (unmodified) residence has a significant and adverse impact on the sustainability of current living and care arrangements [38,39,40,41,42,43,46,47,48,49,50,51,52,53,54,55,56]. The person intends to remain living at the residence (some schemes considered it reasonable that a person stay in the modified home for a specified period of time, unless there were exceptional circumstances) [38,39,40,41,42,43,46,47,48,49,50,51,52,53,54,55,56] The residence is structurally sound and able to be modified safely [38,39,40,41,46,47,48,49,50,51,52,53,54,55,56] Relocation to a more suitable residence is not viable [38,39,40,41,46,47,48,49,50,51,52,53,54,55,56] An assessment by a suitably qualified OT or health professional recommended the home modifications considering all possible alternatives, including the use of equipment or adopting different behavioural techniques [38,39,40,41,46,47,48,49,50,51,52,53,54,55,56] Regarding home modifications to a home purchased after the acquisition of disability: the person is expected to have considered their existing disability needs in the selection of the site and design of the new premises, and that the premises selected provides appropriate access (i.e., any further modifications would be very basic and low cost) [39,50,51,57,58]. Some schemes specifically require appropriate access to the following areas of the new home: one point of access/egress, a bathroom and toilet, a bedroom, a living/dining area, and a kitchen (for people who can fully or partially prepare their own food or beverages) [39,56] Regarding home modifications to rental properties: a written agreement from the owner is required before any modification [41,46,53,56,59,60]. Some schemes require a 12-month lease for modifications to be considered [51].	‘Reasonable and necessary’ home modifications in relation to [37,38,39,40,41,42,43,44,46,50,51,52,56,57,58,59,61,62,63,64,65,66,67,68,69,70,71,72,73,74,75,76,77,78]: private dwellings legacy public and community housing dwellings (although determined on a case-by-case basis and not to the extent that it would compromize the responsibility of housing authorities to develop, maintain and refurbish stock that meets the needs of people with disability) rental properties interim solutions incidental costs associated with modifications (e.g., additional costs incurred for personnel expertise and travel; design and architecture fees; council and other building approvals; home modification assessment, delivery, set-up, and adjustment) modifications specifically targeted toward physical accessibility, bathrooms, kitchens, and spare rooms (on a case-by-case basis with exceptional circumstances)	Modifications that are the responsibility of other parties (e.g., State Government; privately owned residential care facilities; community housing) or systems (e.g., Health systems) [45,47,50,54] Modifications installed in common areas (e.g., rails or paths in blocks of units used by other residents) [45] Fittings, fixtures, or materials that are above standard grade or not deemed reasonable and necessary (how ‘standard grade’ and ‘reasonable and necessary’ is determined was not explained) [50,51,57,58] Cosmetic or personalized fittings not deemed reasonable and necessary [41,49,59,74] In-ground or above-ground swimming pools (including hydrotherapy) and spas [41,50,53,57,58,59,74] Any modification where the need cannot be specifically attributed to the person’s disability [50,57,58] Modifications required based on a pre-existing condition [51,57,59,74] Capital building additions (only funded in exceptional circumstances) [50,58,59] Any loss of value of any home resulting from any modifications to, or removal of modifications from, the home (except with prior agreement in the case of a rental property) [45,50,57,58,59,74] Modifications associated with the natural ageing process of an individual [45] Cost of modifications that exceed the value of the home [57,59] Modifications where the owner, body corporate, or other responsible Authority has not given permission [39,41,51,57,59,74] Modifications to an illegal dwelling or illegal modifications [41,56,59] Repairs and maintenance to a residence as a result of normal wear and tear or general upkeep of the residence, or works not funded by the Authority [39,51,56,66] The connection of basic utilities where they were not connected previously. The connection of utilities will only be funded if an existing utility is changed or moved to enable an alteration to be functional (e.g., the removal of a bathtub and the installation of a hobless shower recess; the grading of a floor; the installation of new hand shower fittings) [54] Significant modifications to a new premises purchased after the acquisition of disability unless the relevant Authority was involved in the decision to purchase the property, or the purchase of a more accessible property was not possible [50,51,56,74] Major modifications to a rental property (unless there is a lease agreement of a predetermined number of years) [59] Additional insurance premiums due to home modifications [50,57]	Some (not all) schemes will fund: costs associated with repairs and maintenance of home modifications due to wear and tear of a property that is a result of the injury (e.g., damage to floorboards from wheelchair use; standard repairs and maintenance to specialized fittings and assistive technology installed) [39,48,49,51,56,57] necessary and reasonable costs of returning a rental property to its former state (if the person moves out of the rental property, and when the costs are related to modifications that were previously approved or installed by the Authority and are related to the person’s injury) [56] reasonable cost of basic access for a secondary residence which is lived in concurrently by the person [39,41,51,56,57] modifications for temporary or casual use [45,56,59] the repair or reinstallation of improperly made or installed modifications (Schemes that do not fund this support consider it the responsibility of the tradesperson or builder) [41,45,50,51] home modifications more than once to a single residence (other schemes will not fund further modifications to the same premises except where there are unforeseen and significant changes to the person’s needs) [39,51,56,57,58].
New builds (owned by individuals)	3 (15%)	The home cannot be reasonably modified due to size, age, condition, design, external terrain and/or value of modifications exceeding the value of residence [41,62,73] The person is expected to have considered their existing disability needs in the selection of the site and design of the new premises [58] Dwellings of all building types must, as a minimum, contain a kitchen, a bathroom, a living/dining area, an entrance/exit, and at least one bedroom [79]	User costs of capital in situations where a person requires an integrated housing and support model and the cost of the accommodation component exceeds a reasonable contribution from individuals [76] Reasonable difference between the cost of building the home and the costs of disability-specific needs (e.g., difference in cost between a standard oven and a disability-specific oven) [41,51] Contribution to the cost of installing a semi-detached unit [41,73]	None identified from publicly available information.	None identified from publicly available information.
Home purchases (owned by individuals)	1 (5%)	The person’s main dwelling must be unable to be reasonably modified [45] and the unit must only include facilities specifically utilized by the person which cannot be accessed or modified within the main dwelling [80].	A reasonable amount toward the purchase cost of a semi-detachable portable unit [62]	A contribution to, or payment of, the full purchase price of a main residence [51].	Not applicable.
Consumables and equipment	19 (95%)	The consumables and equipment requested represent items commonly prescribed by a doctor, physiotherapist or other recognized treatment provider [62,81,82,83,84,85] The items were prescribed on an assessed clinical need related to the person’s injury [39,43,56,61,63,66,69,70,81,82,83,85,86,87,88,89] The items will increase the person’s capacity or safety to participate in an activity [39,42]	Consumables and equipment related to continence; eating, drinking and food/drink preparation; household tasks; personal care and safety; communication and information; building fixtures; clothing and footwear; small stock; equipment intended to treat or stabilize an injury; life support equipment; personal mobility equipment; equipment for sport, leisure, and recreational activities; climate control equipment; assistive technology (including environmental control equipment); disposables; computer technology; lift devices; special food or a special food formula; and the extra cost associated with furniture or appliances adapted or designed to address the person’s functional limitations [36,38,39,40,42,48,49,51,56,61,62,63,64,65,67,69,71,73,75,78,82,83,84,85,86,87,88,89,90,91,92,93,94,95,96,97,98,99,100,101,102,103,104,105,106,107,108] Anything needed to operate, run, maintain, or repair equipment (e.g., electricity, water, lubricating oil, and replacement filters and batteries) [38,51,62,93,99] Incidental costs including assessment, delivery, set-up, adjustment, training, repair and maintenance (due to regular wear and tear), rental costs where necessary/appropriate, and upgrading or modifying equipment that was owned by a person prior to their accident [39,44,48,49,51,61,64,65,67,78,85,89,95,96] In circumstances where the cost of modifying existing equipment exceeds the cost of purchase and the equipment is reasonable and necessary, an Authority may fund the purchase of new equipment [39]	Equipment not related to the person’s injury from the accident [91] Purchase, maintenance, repair or replacement of any household, gardening or lawn mowing consumables, equipment, appliances, or items that are not related to the person’s functional limitations or which would normally be purchased by any person (e.g., general household furniture or appliances), unless recommended by an OT as assistive equipment for the person [39,44,51,56,57,67,88,91,96,106,109,110] Equipment that another agency or department is responsible for providing [56] Equipment that is more expensive than an item that is strictly required to meet the person’s identified needs [39,88] Equipment for areas of the home that the person cannot access or does not access for activities of daily living [100] Cost of insurance for any equipment [106] Further funding to replace an item of assistive technology costing more than a specified amount within a specified timeframe (or the typical service life of that assistive technology if different and indicated at the time of the original funding) [108] The entire cost of energy; or prospective payments for energy costs in advance [38] Cost of equipment associated with firewood (e.g., maintenance of fireplaces; wood heating materials) [95,111]	Some (not all) schemes will fund: partial or full costs for the provision of climate control and home exercise/gym equipment [58,67,78,83,88] necessary and reasonable cost of Internet access, where it is not otherwise available to the person [38,99,112] most schemes fund recreational equipment, except for one (approach currently under review) [78]
Vehicle modifications	18 (90%)	A need for modifications based on a compensable condition [39,56,113,114,115,116,117,118,119] The modifications promote safety, maximize independence, or allow appropriate care [68] The person owns or has access to a vehicle on a regular basis [39,114] The person has an endorsed licence for the vehicle, or has been assessed as having the capacity to obtain an endorsed licence [39,56,114,116,120] If the vehicle is not owned by the participant, written agreement from the owner has been provided [120]	Reasonable changes to a vehicle or the installation of equipment in a vehicle to enable the person to gain access to a vehicle and in some cases operate the vehicle [37,39,43,44,52,56,63,64,68,71,82,115,116,117] Repairs and maintenance of vehicle modifications as these are related solely and directly to disability needs [39,46,56,67,114,116] Incidental costs to support the use of a modified vehicle (e.g., driver assessments for the purpose of obtaining an endorsed license; driving lessons to establish skills to use the modified vehicle or additional lessons where a person’s disability results in them taking longer to learn to drive; assessment, delivery, installation, and adjustment of vehicle modifications; additional insurance costs where an additional insurance premium is payable as a result of the modifications [the Authority will only fund the increased amount of the premium, not the total cost of the policy]; training for use of modifications; the cost of engineering certification and other checks required for initial registration; the costs of obtaining a ‘blue slip’ required for major modifications [State-specific]) [39,48,49,56,114,115,116,117,120] Cost of removal of modifications and reinstallation on a new vehicle when practical and cost-effective [39,56,116] Replacement of the motor vehicle modifications required or installed as a result of the motor accident [39,56,114,120]	Cosmetic or personalized fittings that are not reasonable and necessary (the Authority will fund the reasonable and necessary component of the modification, and the person will pay the additional cost) [49] Non-standard items (e.g., auto docking where the person or their attendant is able to manually dock) [116] Further modifications to the same vehicle except where there are unforeseen and significant changes to the person’s medical condition (it is generally expected that the modifications will be suitable for the person’s anticipated long-term needs) [39,56,114,116] Modifications to a motor vehicle that does not comply with legislation [114,120] Costs to convert a car back to its standard configuration once major modifications have been made [120] Modifications to a car for a person other than the individual with an injury, or that will not be used by the individual [120] Modifications required based on a pre-existing condition [56,114,120] Modifications to a motor vehicle that are of no clear (functional) benefit to the person [114,120] Repairs to adaptive equipment or modifications that are subject to the equipment supplier’s, car modifier’s, or car’s insurance policy [120] Costs associated with registration, regular insurance and petrol [46,56,114,116,120] Maintenance and repairs that all car owners are expected to undertake on their cars in order to keep them in safe working order [120] Driving supervision in order for a participant to accrue hours to pass a driving test [116]	Some (not all) schemes: subsidize the cost of buying a suitable vehicle to meet clinically required needs or provide a contribution of a reasonable amount toward the purchase cost of a suitably modified second-hand car selected by an Authority [39,86,91,114,115,116,118,120] will fund the conversion of a car from manual to automatic transmission [90,114] fund modifications to more than one motor vehicle, if assessed as being reasonable and necessary [39,56,114,115] will fund vehicle maintenance services [39,116]
Short-term accommodation	8 (40%)	The need for accommodation is related to the person’s injury [51,57,121] The person cannot live at home while their home modifications are underway [52,73,121] There is no other existing suitable accommodation option [57]	Costs of short-term accommodation in limited circumstances when a home modification is in progress [39,49,51,52,56,57,73,91] Reasonable costs of short-term supported accommodation services following discharge from hospital or respite care [51] Short-term access to purpose-built group residences to foster independence of residents (with the aim to allow a person who is progressing toward independence the opportunity to do so in a supportive environment prior to moving back into the community) [121,122]	Costs of short-term accommodation when the person’s housing issues existed prior to their injury (e.g., homelessness) [39] Accommodation when a home modification is not in progress [39] Accommodation where the need is not due to the person’s injury [39]	In relation to short-term accommodation while modifications are completed, one (not all) schemes: specified time restraints for the funding provided (e.g., short periods of up to 14 days at a time; a maximum of 6 months) [49,57] will fund short-term accommodation only for the first home modification [39,57] In relation to short-term, transitional accommodation, one (not all) schemes: specified time restraints for the funding provided (e.g., 18 months after first being discharged from hospital or respite care) [51]
Long-term accommodation	6 (30%)	The person requires integrated housing and supports on a permanent or semi-permanent basis due to their injury [51,56,123] The person’s home may be rented, owned, or purchased by an Authority, or it may be an accessible home rented or purchased directly by the person from a housing provider [56] The person may be required to make a reasonable rent contribution or contribute to their daily living expenses [51,56] In relation to specialist disability accommodation (SDA) housing: a specialist housing solution is required [49] the person’s impairment results in an extremely reduced functional capacity to undertake activities of mobility, self-care or self-management, and the person has a very high need for person-to-person supports in undertaking activities even with assistive technology, equipment or home modifications [100] would represent better value for money [55] the SDA provider is registered [101] the person resides in the dwelling, or in a dwelling providing accommodation of the type and in the location (or of a higher-cost type or in a higher-cost location), specified in their plan [101] the parent or parents of the person do not reside in the dwelling [101] at least one private bedroom has been made available for the person or, if the person is a member of a couple, at least one private bedroom and a second room that may be a bedroom or another similar sized private room has been made available to the couple [101] the dwelling is eligible to be enrolled, has been enrolled and continues to be enrolled [101] the number of bedrooms and similar sized private rooms in the dwelling is at least equal to the number of residents for which it is enrolled [101] density restrictions are satisfied where relevant [101]	Permanent or semi-permanent assisted living accommodation [46,49,123,124] In relation to SDA: the cost of the housing (including the land it is on), as well as ongoing costs such as maintenance may be funded [46,49] SDA may include special designs for people with very high needs or may have a location or features that make it feasible to provide complex or costly supports for independent living [46,49] SDA design categories include basic design; improved livability design; fully accessible design; robust design; high physical support design [125] building types may include apartments; duplexes, villas, townhouses; houses; group homes; and larger dwellings [125] people receiving SDA funding could also be eligible for Supported Independent Living (SIL) supports in their package [46,49] individuals with SDA in their plan may pool their SDA budgets and approach a developer to explore having an SDA option purpose-built for them [126]. In relation to Residential Independence Pty Ltd. (RIPL accommodation) [123]: purpose-built, accessible apartments and units across Victoria that integrate assistive technology to assist tenants to live independently access to a client-centred model of support that promotes independence. In relation to supported residential services [127]: a housing model that provides assistance with showering, personal hygiene, toileting, dressing, meals, and medication. Some supported residential services also provide nursing or allied health services. In relation to shared supported accommodation (i.e., group home): houses with paid carers that provide personal care, nursing rehabilitation, housekeeping, meal and laundry services. There may also be a sleepover facility for staff [128,129] reasonable costs for shared supported accommodation services to be provided as a 24 h shared care model [128,129] clinically justified specialist equipment (not defined) in a facility for specific use by a particular client with the consent of the owner of the facility may also be funded [128]. In relation to residential aged care facilities: Funding for hostel accommodation: 24-h supervision for residents. Hostels are staffed by personal care assistants (under nursing supervision) to assist with meals, activities of daily living and medication [127] Funding for nursing home accommodation: 24-h nursing care for residents. Nursing homes are staffed by registered and enrolled nurses, nursing assistants or personal care assistants [127,130]	Most schemes did not report elements relating to long-term accommodation that would not be funded (i.e., it is unknown whether funding caveats exist). Only one scheme specified funding limitations. In relation to SDA: Personal support costs (such as those provided through SIL) that are assessed and funded separately would not be funded [46,49] Accommodation costs where these are not linked to a person’s disability or where specialist accommodation with integrated supports is not required would not be funded [46,49]	None identified from publicly available information.

Note. Data obtained from publicly available information. Since the level of detail provided in each scheme’s public documents and the extent of infrastructure supports funded by each scheme varied dramatically, the information presented in this Table reflects the most accurate representation possible. Additional differences in funding across the schemes may be identified if/when further details about funded infrastructure supports are provided by each scheme.

**Table 3:** A Comparison of Tenancy Funding across the Schemes.

Subcategory	No. Schemes Funding Available	Eligibility Criteria	Description of Funded Supports	Description of Supports Not Funded	Differences across Schemes (if Applicable)
Rent assistance	1 (5%)	Home modifications are underway [51]	Short-term rent (period of time not specified) [51]	Long-term rent assistance (period of time not specified) [51]	Not applicable.
Support obtaining and/or maintaining a tenancy	7 (35%)	In relation to obtaining a new tenancy: The person’s home is unable to be reasonably modified (including rental properties), or relocation is the most appropriate option [39,51] The person is unable to look for alternative properties by searching the Internet or liaising with real estate agents, or does not have family or friends to assist them to locate a suitable property [39,56,57,74]	In relation to obtaining a new tenancy: Necessary and reasonable costs of assistance to locate a suitable property for purchase (e.g., an assessment by an OT or an appropriately qualified person) [39,41,46,51,56,57,74,75] Assistance in applying for a rental tenancy (type of assistance not specified) [49] In relation to maintaining an existing tenancy [49,76,131]: Support to build a person’s capacity to maintain their tenancy (e.g., undertake tenancy obligations in line with the person’s tenancy agreement) Support for appropriate behaviour management	Homelessness-specific services including homelessness prevention and outreach, or access to temporary or long-term housing for people who are homeless or at risk of homelessness [76] Cost of Internet to research suitable properties [57]	None identified from publicly available information.
Relocation costs	6 (30%)	A home cannot be reasonably modified due to size, age, condition, design, or external terrain [39,41,50,57,59,62,73] The home is unable to be cost-effectively modified (e.g., the value of modifications exceed the value of the residence) and relocation is the most appropriate option [39,41,50,56,57,59,73,74] A person who chooses to move into another home must take their injury requirements into account [41] The person is moving into a suitable home or a home that is capable of being reasonably modified [51,62] The person will locate a property that does not require substantial modification [56]	Reasonable contribution to the cost of relocating the person to another home [41,62,73] In relation to private properties: [39,50,51,56,57,59,74] reasonable costs associated with a house sale the cost of one strata, building, or pest inspection report bank fees limited to the relocation process interim rental accommodation reasonable cost of removalist services if the person is required to move as a direct result of their injury or illness (full or partial cost, depending on what is considered reasonable) In relation to rental properties (where a person is relocating from a rental property to another rental property): [51] reasonable costs payable for the requirement to break the tenancy agreement early reasonable cost of removalist services if the person is required to move as a direct result of their injury or illness (full or partial cost, depending on what is considered reasonable)	The cost of more than one strata, building, or pest inspection report [39,57,74] Costs of any repairs or maintenance issues identified in strata, building or pest inspection reports [39,57,74] Body corporate/strata fees [39,57,74] Home insurance [74] Council or water rates [39,57,74] Moving costs if the person decides to move house for reasons unrelated to their injury [74] Packing and unpacking boxes as part of moving house [121] Other costs associated with the end of a tenancy that are a condition of the lease (e.g., advertising costs associated with breaking a lease, steam cleaning of carpets or cleaning a property at the end of a tenancy) [56]	None identified from publicly available information.

Note. Data obtained from publicly available information. Given the limited detail provided in each scheme’s public documents about funded tenancy supports, the information presented in this Table reflects the most accurate representation possible. Additional differences in funding across the schemes may be identified if/when further details about funded tenancy supports are provided by each scheme.

**Table 4:** A Comparison of Service Delivery Funding across the Schemes.

Subcategory	No. Schemes Funding Available	Eligibility Criteria	Description of Funded Supports	Description of Supports Not Funded	Differences across Schemes (if Applicable)
Attendant (personal) care	17 (85%)	A demonstrated need due to permanent or long-term eligible injuries, and the person’s need is recognized by a medical practitioner [36,37,51,62,64,71,81,132,133,134,135,136] The support required by the person is not the usual responsibility of the Health System [132] The request for support meets specified hours per day (where applicable) [132] For a support worker to be employed to sleep overnight, they must be provided with a separate room with a bed and the use of facilities (e.g., bathroom) [137] Support requested for injured children does not replace the usual care and supervision provided, or paid for, by a parent [132] Support for injured children may be funded when the level of support needed is beyond the level usually required for children of the same age [132] For one scheme, support for injured people with caring responsibilities only available where the person lived with and provided care to a member of their immediate family before the accident, and they continue to live with the person afterwards [136] For on-call support, the person has a demonstrated need for the service (e.g., they live alone or with people who cannot provide assistance), capacity to operate a personal alarm, is not at risk of a medical emergency that may prevent them from using the alarm, has identified independent living as a goal, requires minimal hours of assistance (e.g., minimal or no assistance needed during the day and/or through the night), and the person’s need for care can be reduced with the provision of appropriate equipment [95] One scheme specified eligibility for post-acute support when the person requires treatment in hospital as an inpatient or for day surgery more than three years after their accident (period of time post-acute support provided not specified) [138] One scheme specified that in the case of a person requiring daily personal care, the total daily or weekly expenses payable are not to exceed the total daily or weekly expenses payable if the person was being provided with attendant care and domestic services in available purpose-built group accommodation [43]	Self-care activities (including personal hygiene; toileting, bladder and bowel management and menstrual care; eating and drinking; taking medication; putting on compression stockings, protective bandaging, splints, callipers, and basic first aid; use of aids and appliances, hearing and communication devices; mobility and transferring) [36,37,39,40,42,48,49,64,67,71,75,81,82,87,90,91,92,93,94,110,127,134,135,136,139,140,141,142,143,144] High level clinical support (e.g., managing oxygen or a ventilator, complex wound management, complex continence management or palliative care needs) [145] Cognitive and behaviour support [145] Services may be daytime support or overnight, on-call, or shared support [51,120] Attendant care services may be provided for a short period (e.g., post-acute care; overnight support), for a longer period to meet ongoing needs or for an interim period where injury has not stabilized sufficiently to assess if long-term attendant care is required [110,135,137,138,140,141] Attendant care services may be required across a variety of settings (e.g., a person living alone in their own home; living with family or other people; when undertaking social, recreational, education or employment activities; or during holidays away from home) [132] Personal care supports for children with complex needs [132,136] Necessary and reasonable attendant care services to assist an injured person to carry out parenting or carer responsibilities [136]	Supports funded by other areas of government, including school teacher aides and hospital and GP visits [146] Services not considered essential or reasonably required because of the person’s injury [140] Services for an injury, condition or circumstance that existed before the person’s accident or that are not a result of the accident [94] Services that are of no clear benefit to the person [94] Services for other members of the person’s family or household [94] Post-acute support is not available where childcare, home services, or income support are already being received [138] In relation to attendant care services for injured children: Direct care or supervision to any other siblings or children [136] Services in place of ordinary parenting duties, or for age-appropriate services that parents ordinarily use (e.g., babysitters, nannies, childcare costs or out-of-school-hours care) [39,132,136] The presence of an attendant care worker to meet care needs related to the child’s injury does not replace parental responsibility to supervize and provide non-injury related care to the child [56]	Some (not all) schemes will fund attendant care accommodation, attendant care sleepover, sleepover facilities for staff, and attendant care travel [67,127,140]
Household services	17 (85%)	Supports reasonably required due to the person’s injury, at the request of the person’s treating doctor or health professional that confirms the person needs help for the specified period of time [37,42,43,62,64,71,81,110,111,135,136,139] The person performed the tasks before their injury [42,43,51,71,95,147] There are no household members able to provide this support [49,147] One scheme specified that where an injured person who is entitled to household services employed another person to carry out household duties before the date on which the person was injured, the injured person is entitled to recover only the cost of those duties that are additional to those carried out by the housekeeper before that date [43] Documentation of the support needs of an injured child, for tasks ordinarily provided by a parent or family member as part of their parental responsibilities, must include a description of why the assessed needs of the child require the assistance of a support worker [56] Household services for injured people with caring responsibilities will only be available where the person lived with and provided care to a member of their immediate family before their accident, and they continue to live with the person afterwards [136] Some schemes specified time restraints (e.g., payable up to 40 h per week in total, for up to 5 years after the accident). However, individuals with severe injuries and/or permanent disability from the accident may receive support at home after this period with approval. A letter from the person’s treating doctor or health professional that confirms the person needs help for that period of time is required [42,43,147]	Services of a domestic nature required for the proper running and maintenance of the person’s household (e.g., internal house cleaning; bill paying; unaccompanied shopping; seasonal and occasional household tasks; bushfire readiness) [37,39,40,42,43,49,51,64,67,69,70,75,81,82,86,87,90,91,94,95,110,127,135,136,139,144,147,148,149,150,151] Household services can be provided on a short-term basis (e.g., while the person is recovering from surgery) or for a longer period to support the person’s ongoing requirements [148] Necessary and reasonable support services to assist an injured person with parenting or caring responsibilities to carry out their responsibilities [136] In relation to firewood provision: Short-term assistance with firewood up to a specified maximum amount [51] If long-term assistance is needed, an Authority may pay up to the equivalent of the cost of assisting the person to obtain firewood for a specified maximum time period (e.g., 3 years) and maximum cost towards a reasonable alternative heating option [51] In relation to household services for children with injury: necessary and reasonable expenses of support or domestic services in place of attendant care services in order to allow the parent to meet a care need that is related to the child’s injury [136]	Services which a person could not reasonably have performed for themselves prior to their accident [95,111] Services provided by a provider that is not registered and approved by the Authority [95] Services provided by telephone or other non face-to-face mediums, including telephone calls and telephone consultations between providers and clients and between other providers [95] Services provided more than once on the same day to the same person [95] Services provided by friends or family members [95] Services that extend to other adults in the home [95] The cost of food, pet care, or car care [49,95,152] Regular household maintenance (e.g., painting; decorating; house repairs; plumbing; electrical work; and cleaning of drapes, blinds, or carpets) [95,148,153] Tasks the person received a salary or wage for before the accident [111] Household services for a person absent from their primary residence for reasons other than a hospital admission due to their accident injuries [111] Major repairs or services requiring a qualified tradesperson [110] Fees for services such as waste removal or tip fees [95,110,111] Domestic services for a person living in a residential facility [111] Household tasks that the person is able to do themselves, or that their family members can reasonably be expected to help with [147] In relation to firewood provision and heating alternatives: The cost of State Forest licences required to collect firewood or permits which the person did not hold before the accident [95,111] In relation to household services for children with injury: services in place of ordinary parenting duties, or for age-appropriate services that parents ordinarily use (e.g., babysitters, nannies, childcare costs or out-of-school-hours care) [94,136]	Some (not all) schemes will fund: routine, cosmetic, or ornamental gardening services (e.g., weeding, maintaining flower beds, regular lawn mowing, tiding edges, and sweeping paved areas). However, some schemes will only fund lawn mowing and pruning if an environmental health or safety hazard exists. For these schemes, routine, cosmetic or ornamental gardening services will not be funded [79,84,85,86,88,94,110,121,127,131,133,136,137,140,151,152,154,155] full preparation and delivery of meals, whereas other schemes will only fund assistance with meal preparation [46,84,86,88,94,110,121,131,133,136,137,151]
Community access and participation	11 (55%)	Individuals have a compensable injury and incur extra carer expenses while undertaking reasonable social, recreational, community or disability-related activities [39,51,154,156,157,158] Support need is related to the person’s disability and assists the person’s goals, objectives and aspirations [39,46,48,49,51,92,93,158] Support need is directed by a recommending OT [155] Requested support alternatives are age appropriate [39] Requested Community Group Programs are run by approved disability services organizations [158] One scheme specified that a leisure option offers a genuine alternative to attendant care for recreation and/or community access. The leisure option will therefore replace some of the person’s attendant care services in meeting their needs for community access and recreation. If a person’s needs or circumstances change and they decide to withdraw from a leisure option, their support needs and funding of attendant care, or like responses, will be reviewed and the person’s services adjusted accordingly [159] In relation to transport, individuals may receive higher funding to enable their participation in employment [160,161]	Attendance at reasonable social, recreational, community, or disability-related activities, including travel to these activities. Funding may include: [39,46,49,51,67,94,102,136,143,154,155,156,157,158,159,162,163] Attending social, community or recreational activities, such as visits to the movies or sporting matches Less frequent events such as attendance at significant family event (e.g., wedding, funeral, holiday season gatherings) Attendance at an interstate appointment where such a service is not available locally Attendance at a reunion or significant event in the community, to which the person belongs or closely identifies with Personal assistance to participate in recreation activities (e.g., changing into sports clothes, manipulating equipment, positioning to undertake the activities) Engagement in activities with capacity building components to build skills and independence Group-based community, social and recreational activities Group-based social and recreational activities in a centre-based program In relation to companion (relative and service provider) support: carer meals and admission tickets to activities may be funded [67,154,158,164] In relation to community-based grants: funding to organisations, groups, and private companies for activities and services that improve the health and wellbeing of people with disability [165] In relation to community awareness initiatives, funding may be available to: Assist organisations to adjust to the specific needs of their member with an injury where that adjustment is not part of their universal obligations under reasonable adjustment [166] Support organisations to increase their awareness of the needs and desires of people with disability, and explore strategies to assist them to address simple improvements that may be needed to their facilities to enable access and participation by people with disability [167] In relation to transport: [49,67,75,158,160,161,164,168] Travel for the person with disability (not otherwise specified) Supports that enable the person to build capacity to independently travel Reasonable and necessary costs of taxis or other private transport options for people not able to travel independently Transport assistance (not specified) if the person cannot use public transport without substantial difficulty due to their disability and it is not reasonable to expect that family or the community would provide the transport Travel to attend community group programs. Individuals who are independent in driving or public transport use may be able to claim mileage, or met tickets whilst attending an approved disability support program.	Entry fees, activity costs, or cost of materials [46,61,152,154] Equipment hire and/or hire of facilities [61,156,158] Teaching or instruction of the activity [61,156,158] Gym memberships [46] Hobbies or personal lifestyle interests (e.g., hobby farm; animal breeding or showing) [95] In relation to transport: Travel costs for anyone other than the person (unless they have been pre-approved to be accompanied by a companion) [164] Modifications to public transport or taxis [160] Ensuring that public transport options are accessible to a person with disability, including through the funding of concessions to people with disability to use public transport [131,161] Compliance of transport providers and operators with laws dealing with discrimination on the basis of disability [131,161] Transport infrastructure, including road and footpath infrastructure, where this is part of a universal service obligation or reasonable adjustment [131,161] Support to compensate for the lack of a public transport system [131,161] Travel costs where the person chose to use a mode of travel over and above what the Authority considers necessary and reasonable [164]	In relation to transport: Four schemes will not fund the person’s travel costs except to and from treatment and rehabilitation services where expenses are paid by the Authority [39,94,164,169] While four schemes stated they will fund companion travel [67,68,154,164], one scheme will not fund travel expenses for companions for ‘normal daily activities’ (e.g., travel to work, shops, or social functions with the person) [164]
Case management services	4 (20%)	Person has a disability as a result of transport or workplace accident injuries, and requires additional support [124,158,170] Services are considered reasonable and necessary [171]	Case management services may assist with [124,130,158,170,171,172,173,174] Making informed decisions and exercising choice and control Arranging any assessments required to access funding Negotiating services and prices with preferred providers Strengthening and enhancing the person’s capacity to coordinate, self-direct, and manage their supports Facilitating transition planning (e.g., finding suitable accommodation; transitioning from a secure environment into the community) Overseeing and ‘walking’ the person through complex legal matters such as custodial issues or criminal charges (legal matters must not relate to legal disputes with the Authority) Supporting the person to re-establish their role in the community, at home, in school, at work and in leisure activities Monitoring the person’s participation in community activities to ensure programs continue to appropriately address the person’s needs Supporting the person in their reunification with significant others, and maintaining and enhancing peer support networks Proactive contingency planning and, where necessary, crisis intervention Ensuring the person is receiving all benefits and entitlements from the Authority that they are entitled to Referring the person to Centrelink to check eligibility for benefits Referring the person to counselling services Facilitating sharing of Agent-funded support services Linking to mainstream services (i.e., public housing, education, transport, health, aged care) Connecting people with disability, their families, and carers (including people who are not participants in a particular scheme) to disability and mainstream supports in the community Assisting the person to prepare for any reviews of their plan Providing the reasonable travelling expenses necessary for a case manager to complete assessments and provide ongoing support	Only one scheme specified case management services they would not fund: [171] Plan administration or management Support rostering Advocacy Disability supports (not specified)	One scheme will fund advocacy support while at least one scheme does not [170,171]
Family supports	11 (55%)	In relation to respite services: it can be demonstrated that respite will enhance the functioning of the family unit and enhance sustainability of the regular care or support routine [39] respite services may be provided to someone who is a carer of an entitled person; an entitled person who is a carer; or a self-carer (otherwise not defined; for residential respite only) [110] In relation to specialist behavioral intervention support: the person displays significantly harmful or persistent behaviours of concern [175] In relation to training: the training will assist the person and their family to achieve greater independence and/or cohesion, and it represents a cost-effective option [136] In relation to people with injury who have caring responsibilities, to access childcare services: the person cared for a child or children before their accident (or was pregnant at the time of their accident) and is now unable to care for them due to their injuries [51,147,176,177] there is an absence of other community and/or family support that might reasonably be expected to provide childcare services [51,176,177] Requested childcare services are age appropriate, provide appropriate support and are assessed as a suitable alternative to meet the person’s injury-related needs [39]	Supports that families need as a result of a family member’s disability, as well as supports that enable sustainable informal caring by family members and friends. These supports may include: [75,81,173,175] family support and counselling due to a family member’s disability building the skills and capacity of other family members to manage the impact of a person’s disability on family life supports that increase the person’s independence, as well as supports that enable the person to enjoy social and community activities independent of their informal carers In relation to respite services: [39,67,82,91,110,122,129] reasonable and necessary costs of in-home respite care, residential respite care, or Emergency Short-Term Home Relief In relation to specialist behavioral intervention support: [175] highly specialized intensive support interventions to address significantly harmful or persistent behaviours of concern the development of behaviour support plans that temporarily use restrictive practices, with intention to minimize use of these practices In relation to training: [136,175] training for immediate family members or people who live with the person in matters related to caring for a person with disability (particularly when equipment, medical aids, or manual handling may be required) training for carers and others in behaviour management strategies required due the person’s disability In relation to people with injury who have caring responsibilities: specialist assistance in the home (not defined) may be provided to strengthen the sustainability of informal supports [175] reasonable costs of childcare services (i.e., funding for the amount of time that the person would have been providing care for the child or children but is now unable to do so due to the accident) [51,176,177] when the parent is unable to access or secure a place in approved childcare, an Authority may pay for registered childcare such as a nanny or au pair [177]	Supports that are funded under the community services system, even if the system does not provide them. Community services system includes: [175] general family support and counselling, parenting skills programs, and family relationships services all aspects of the statutory child protection system arranging out of home care for children subject to child protection orders (including making these arrangements sustainable for children with disability) guardianship arrangements for people under the age of 18 years In relation to people with injury who have caring responsibilities, childcare that: [147] is performed by family and friends (except for registered childcare) is not related to the accident injuries not done personally by the injured person before the accident (unless the person was pregnant at the time of their accident)	In relation to respite services, only one scheme specified a limit (i.e., number of hours or days in a calendar year) that an eligible person may apply for respite [110] In relation to an injured child: Some (not all) schemes will fund reasonable expenses of school holiday programs or childcare [39,56,127,136,138,144,147] In relation to people with injury who have caring responsibilities, only one scheme specified the following eligibility criteria to access childcare services: the person requires childcare services in order for them to receive or attend medical or rehabilitation treatment for their injury [51,176] to access registered and in-home childcare services specifically, all other forms of childcare have been explored and deemed inappropriate and the family lives in a rural or remote location [51,176]
Assistance animals	4 (20%)	Required as a result of accident injuries [178]	Suitably trained Guide and Assistance Dogs for people with visual or hearing impairments. Funding may include the dog, harness, training, freight, accommodation during training, and veterinary costs when needed [136,179]	The use of an assistance animal to restrain a person outside of an approved behavioural support plan [180] Feeding, grooming and general day-to-day maintenance costs of an animal [178,180] Ongoing veterinary costs once the animal has retired from service [178]	One scheme will not fund: the purchase, transport, or upkeep of dogs to assist with the management of symptoms of mental health conditions such as PTSD, anxiety, or depression “until sound evidence exists” [181] additional training (animals provided are expected to be fully trained) [178]

Note. Data obtained from publicly available information. Since the level of detail provided in each scheme’s public documents and the extent of service delivery supports funded by each scheme varied dramatically, the information presented in this Table reflects the most accurate representation possible. Additional differences in funding across the schemes may be identified if/when further details about funded service delivery supports are provided by each scheme. GP = General practitioner; PTSD = post-traumatic stress disorder.

**Table 5:** Summary of Demographic Factors That Influenced the Provision of Housing-Related Funding.

Demographic Factor	Description
Age	The person’s current age and age at the time of injury [137]
	For children with ABI or SCI [39,56,94,132,136]: There is an expectation that parents will provide substantial care and support for children; Funding may be provided if the child’s care needs are due to their disability and substantially greater than those of other children of a similar age (note that scheme authorities will consider what is reasonable support for parents and other family or community members to provide in light of the support parents have to provide the child generally, even with disability); The extent of any risks to the wellbeing of the child’s family members or carer/s (how risks are defined and assessed not explained); and Whether the funding or provision of the support for a family would improve the child’s capacity or future capacity (i.e., independence), or would reduce any risk to the child’s wellbeing (how independence and risks are defined and assessed not explained)
	For adults with ABI or SCI [76]: The extent of any risks to the wellbeing of the person arising from the person’s reliance on the support of family members, carers, informal networks and the community (how risks are defined and assessed not explained); The suitability of family members, carers, informal networks and the community to provide the supports that the person requires (e.g., age and capacity of the person’s family members and carers, including the extent to which family and community supports are available to sustain them in their caring role); The intensity and type of support that is required and whether it is age appropriate for a particular family member or carer to be providing that care (how this is assessed not explained); The extent of any risks to the long-term wellbeing of any of the family members or carers (e.g., a child should not be expected to provide care for their parents, siblings or other relatives or be required to limit their educational opportunities; other risks not defined); and The extent to which informal (unpaid) supports contribute to or reduce a person’s level of independence and other outcomes (how this is assessed not explained)
Gender	Whether supports provided are gender appropriate for a particular family member or carer to be providing that care [76]
Location	Price limits differed across Australian states/territories [49]
	Urban/regional/rural areas: Price limits differed between urban and remote/very remote areas (to accommodate additional service delivery costs) [49,51,176] Available wheelchair accessible public transport in the person’s local area (e.g., buses, trains) or any public transport (e.g., limited in regional areas and often no public transport in rural areas) [137]
Culture	Recognition that a person’s cultural background may influence their choices for supports [137], but no explanation of how schemes actually respond to different cultural needs
Degree of functioning	The person’s diagnosis, progress of recovery, and prognosis [39,43,56,58,61,63,66,69,70,81,82,83,85,86,87,88,89,112,148]
	Specific functional limitations (e.g., height; weight; upper and lower limb function; posture; balance; cognitive, communication, behavioural or emotional issues resulting from the person’s injury) [36,37,38,39,40,41,42,43,48,49,51,56,61,62,63,64,65,66,67,69,70,71,73,75,76,78,81,82,83,84,85,86,87,88,89,90,91,92,93,94,95,96,97,98,99,100,101,102,103,104,110,126,127,131,134,135,136,139,140,141,142,143,144,145,159,175]. How these limitations are assessed were not explained.
	Person’s level of function regarding transfers, mobility, pressure management, personal care, domestic tasks, community access and engagement in work/recreation/leisure activities [36,37,38,39,40,41,42,43,46,47,48,49,50,51,52,53,54,55,56,57,58,59,60,61,62,63,64,65,66,67,69,70,71,73,75,78,81,82,83,84,85,86,87,88,89,90,91,92,93,94,95,96,97,98,99,100,101,102,103,104,110,126,127,134,135,136,139,140,141,142,143,144,154,156,157,158,159,160,161]. How these tasks are assessed were not explained.
	Whether the person has reached maximum medical improvement [182]. How this is assessed was not explained.
Intrinsic characteristics	The person’s attitude, motivation, perceived control in life, positive self-image, coping style, and adjustment to disability may influence their personal resources and thus influence their need for support [112,137]
Whether the person is in receipt of external compensation	If the person is in receipt of external compensation which is intended to be used to pay for supports of a kind which a scheme would ordinarily fund, the compensation is taken into account when determining the type of supports and amount of funding the relevant scheme will provide [38,76]
Whether the person has a pre-existing medical condition or comorbidities	If the person’s medical condition or comorbidities are pre-existing, funding will be the responsibility of another party (e.g., Health system) [38,50,57,58,59,74,87,94,159]. If the person’s medical condition or comorbidities are disability-related (i.e., result of the person’s accident), housing-related supports may be funded by the relevant scheme [36,37,51,58,62,64,71,81,132,133,134,135,136].

Note. Data obtained from publicly available information. Since the level of detail provided in each scheme’s public documents and the extent of housing supports funded by each scheme varied dramatically, the information presented in this Table reflects the most accurate representation possible. Schemes may differ in how demographic factors influence the provision of housing-related funding. These differences may be identified if/when further details about funded service delivery supports are provided by each scheme.

## Appendix A: Overview of Major Australian Funding Schemes (as at June 2018)

**National Schemes**

At a national level, the National Disability Insurance Scheme (NDIS), administered by the National Disability Insurance Agency (NDIA), provides lifetime support for eligible Australian residents under the age of 65 years with permanent disabilities, their families and their carers.

Several national workers compensation schemes also exist. Seacare, which is overseen by the Seafarers Safety, Rehabilitation and Compensation Authority, provides workers compensation to seafaring employees and appropriate third parties. Comcare is a no-fault workers compensation scheme that provides a range of payments and supports to eligible employees of the Commonwealth Government agencies and statutory authorities, the ACT government and corporations or authorities who have been granted a licence to self-insure. Current or former members of the Australian Defence Force with injuries caused by their military service may also be eligible for compensation under the Military Rehabilitation and Compensation Act 2004, including income payment for periods in which they are unable to work, payments for medical treatments and rehabilitation, and permanent impairment compensation.

**State/Territory-based Schemes**

Queensland

In Queensland, people who sustain a serious injury in a motor vehicle accident may be eligible to receive necessary and reasonable lifetime treatment, care and support under the National Injury Insurance Scheme Queensland (NIISQ), which is implemented by the National Injury Insurance Agency Queensland. This no-fault scheme complements the existing Compulsory Third Party (CTP) scheme, providing support to people who may not have been eligible for support previously under the fault-based CTP scheme. People who sustain a serious injury resulting from a motor vehicle accident who are eligible to make a claim through the CTP scheme may receive compensation for general damages such as pain and suffering or economic loss, in addition to lifetime treatment, care and support through the NIISQ. The Queensland workers compensation scheme, WorkCover Queensland, will also engage the National Injury Insurance Agency Queensland to manage the treatment and support of eligible people who sustain serious injuries resulting from a workplace accident.

New South Wales

In New South Wales, the Lifetime Care and Support Authority (part of icare) is responsible for administering the no-fault Lifetime Care and Support Scheme, which funds treatment, rehabilitation and care for people who have sustained a severe injury as a result of a motor vehicle accident or workplace accident in New South Wales. Under the CTP scheme, people may be eligible for income and medical benefits regardless of fault, and people with serious injuries may also be eligible to claim compensation for economic or non-economic loss if they are able to prove that someone else was responsible for the accident that caused their injury. icare also provides workers insurance, and those who sustain severe injuries in the workplace may be eligible to receive payments through the icare Workers Care program.

Australian Capital Territory (ACT)

In the ACT, people who sustain a catastrophic injury as a result of a motor vehicle accident or workplace accident may be eligible for the ACT Lifetime Care and Support Scheme. This is a no-fault insurance scheme that provides eligible participants with long-term care and support. People seriously injured from a workplace accident may also be eligible for workers compensation for economic or non-economic loss, not including future medical expenses or support, which would be covered by the Lifetime Care and Support Scheme. Similarly, individuals injured in a motor vehicle accident and able to provide evidence that another person was at fault may be able to make a CTP claim for compensation for economic loss (e.g. lost wages) and non-economic loss (e.g. pain and suffering).

Victoria

In Victoria, the Transport Accident Commission is responsible for the no-fault Transport Accident Compensation Scheme, which covers long-term services and supports for people who have sustained serious injuries. Through the workers compensation scheme, WorkSafe Victoria, people who are seriously injured in workplace accidents may be eligible to receive payments for lost wages and superannuation, expenses for treatment related to their injury, permanent impairment benefits and may be able to claim for damages from their employer.

Tasmania

In Tasmania, people who have been seriously injured in a motor vehicle accident may be eligible to receive treatment and support, including long-term care, through the no-fault Motor Accident Insurance Scheme administered by the Motor Accidents Insurance Board. Through WorkSafe Tasmania, people who are seriously injured in a workplace accident (regardless of fault) may be entitled to receive weekly payments while unable to work, compensation for necessary and reasonable medical, rehabilitation and other expenses related to their injury, lump sum compensation for permanent impairment, and depending on fault may seek common law damages.

Northern Territory

In the Northern Territory, the Motor Accidents Compensation Scheme is a no-fault insurance scheme that provides a range of treatment and support for eligible people injured in motor vehicle accidents. People who are injured in a workplace accident may be entitled to compensation for weekly payments while unable to work, reasonable hospital, medical and rehabilitation treatment, and payments for permanent impairments.

South Australia

South Australia’s no-fault Lifetime Support Scheme, administered by Lifetime Support Authority, provides treatment, care and support across the lifetime to individuals who sustain serious injuries as a result of a motor vehicle accident. Adults injured in a motor vehicle accident may be eligible for compensation through the CTP scheme if they are able to prove that their injuries are the fault of another driver. For people who are under the age of 16 when the injury occurs, compensation is available through the CTP scheme for treatment, care and support regardless of fault. ReturntoWork SA manages workers compensation claims in South Australia, providing income support and lifetime care and support for people who are seriously injured in a work accident.

Western Australia

In Western Australia, the Insurance Commission of Western Australia manages both the Catastrophic Injuries Support Scheme and the CTP scheme, which both provide lifetime care and support to eligible people who are injured in a motor vehicle accident. However, the CTP scheme is fault-based, so only people who can prove the fault of another driver are eligible for support through this scheme. Individuals must be unable to claim in the CTP scheme to be considered eligible for the Catastrophic Injuries Support Scheme. Compensation for economic or non-economic loss may also be claimed through the CTP scheme. Those seriously injured at work may be eligible for workers compensation through WorkCover WA, including compensation for loss of wages, reasonable medical, allied health treatment and workplace rehabilitation expenses, and some travel expenses related to the injury treatment.
